# A scoping review on the methods of assessment and role of resilience on function and movement-evoked pain when experiencing a musculoskeletal injury

**DOI:** 10.1186/s12891-022-06058-2

**Published:** 2022-12-15

**Authors:** Elise M. Robinson, Peter J. Clothier, Helen Slater, Amitabh Gupta

**Affiliations:** 1 Western Sydney University, School of Health Sciences, PO Box 1797, Penrith, NSW 2751 Australia; 2 Curtin University, Curtin School of Allied Health, enAble Institute, Bentley, Australia

**Keywords:** resilience, musculoskeletal, injury, movement, pain, function

## Abstract

**Background:**

Resilience refers to an individual’s ability to maintain effective functioning, by resisting, withstanding or recovering from stressors or adversity, including pain associated with physical injury (J Clin Psychol Med Settings 28:518–28, 2021). The aim of this scoping review is to determine the role of resilience in the experience of movement-evoked pain (MEP) and return to functional activity following a musculoskeletal injury.

**Methods:**

This review conformed to the Preferred Reporting Items for Systematic Reviews and Meta-Analyses Extension for Scoping Reviews and the scoping review protocol of the Joanna Briggs Institute (JBI). Five databases and one grey literature database were searched using predetermined key words and index terms to capture published and unpublished records on the topic. Two authors independently screened the title and abstract of each record, with the full-text of eligible records being reviewed. Papers were eligible for inclusion if they examined the population, concept and context of interest, were written in English and the full text was available. Data were extracted from each eligible record to guide discussion of the available literature on this topic.

**Results:**

Of 4771 records, 2695 articles underwent screening based on their title and abstract. After title and abstract screening 132 articles were eligible for full text review, with 24 articles included in the final analysis. This review identified that psychological resilience has primarily been investigated in the context of a range of age-related pathologies. The choice of functional and movement-evoked pain assessments in the included studies were often guided by the pathology of interest, with some being general or injury specific.

**Conclusion:**

This scoping review identified inconsistent conclusions regarding the role of resilience in the experience of MEP and the ability to return to function for older adults with a musculoskeletal injury. This scoping review highlights the need for longitudinal research to be conducted that allows a broader age range, including younger adults, to determine if multidimensional resilience may promote recovery form musculoskeletal injury.

**Supplementary Information:**

The online version contains supplementary material available at 10.1186/s12891-022-06058-2.

## Background

Pain is a complex and subjective experience common to most humans. Pain is considered the fifth vital sign and a perception present as early as the first month of life [1]. Over 1.7 billion people live with a musculoskeletal condition [2] and it is estimated that lower back pain and other musculoskeletal disorders account for 4.1% of all Disability-Adjusted Life Years (DALYs) for all age groups [3]. Musculoskeletal conditions and injuries relate to those that arise from damage or trauma to bones, muscles, ligaments, joints or connective tissue [4]. Musculoskeletal conditions can vary in their mechanism, presentation and duration, and are characterised by the report of pain. Depending on the severity of the condition or injury, a person may experience acute pain that resolves quickly or endures [5].

People with a musculoskeletal condition are often able to continue with activities of daily living (ADL) even while experiencing pain [6]. Movement-evoked pain (MEP) or activity-related pain, refers to the experience of pain during locomotion, movement, activities of daily living and physical activity, rather than pain that is experienced at rest [6, 7]. Musculoskeletal pain varies from other types of pain as it is experienced at the site of peripheral musculoskeletal structures and is said to be intense and localised [8]. Through the integration of sensory, motor and psychological inputs, movement is often the primary stimulus for its experience [9]. Typically driven by peripheral mechanisms, MEP varies from central sensitisation where pain is amplified, pain may be widespread and peripheral pathology may not be present [10].

The assessment of MEP has great clinical utility compared to assessments that investigate pain at rest, as they allow clinicians to identify functional impairments related to pain and can guide exercise rehabilitation, facilitate goal setting and predict future disability and pain [6, 11]. When experiencing acute pain, there may be local inflammation and pain at rest [7], which can limit the ability to associate a change in symptoms with a change in function [12]. In contrast, the link between chronic musculoskeletal pain and function is more variable. There may or may not be tissue damage and nociception [12, 13], however, understanding the relationship between MEP and function remains important to inform care and move people back to valued activities.

The ability to remain active when experiencing acute, chronic or recurrent pain is complex and influenced by a number of biological, psychological, social and cultural factors [6]. While acute pain has an important protective role, this role is often unhelpful when tissue recovery has occurred and pain persists. Resilience is a multidimensional construct which describes an individual’s ability to maintain effective functioning by resisting, withstanding or recovering from stressors or adversity, including pain associated with physical injury [12, 14]. Consideration of risk and resilience mechanisms may better explain why some individuals are able to function well with little disability in the presence of severe pain, whilst others with lower pain severity continue to experience significant disruption to daily function [14]. It is possible that resilience may mediate or moderate associations between musculoskeletal pain and function, with this relationship needing analysis to enhance our understanding of movement and pain [6]. In this context, it is important that the role of resilience is understood to guide targeted care especially for those with low resilience who experience high levels of disability, or are at risk of chronicity and poorer outcomes [15].

There is some ambiguity in the definitions used, with traits, trajectories and outcomes all being used to conceptualise resilience. The term resilience has been used in a number of contexts in the literature, including health, emotional, psychological, dispositional, pain and physical resilience [16]. These types of resilience can be considered in the context of MEP in order to better understand how people manage and recover from injury. Psychological resilience describes the range of individual and environmental resources that enable individuals to adapt and recover after adverse or stressful events [17]. A number of psychological contributors to resilience have been extensively investigated, with findings suggesting that optimism, hope, positive affect, self-efficacy and pain acceptance are associated with positive outcomes when coping with back pain [18]. Physical resilience is a relatively new and emerging construct and defined as a person’s ability to prevent functional decline in response to a stressor or recover their physical health after a stressor [16, 19]. Similarly, pain resilience describes a person’s ability to maintain helpful psychological and physical functioning when exposed to persistent pain [20]. It is apparent that various types of resilience may be interlinked and need to be examined collectively rather than in isolation due to the multidimensional nature of musculoskeletal pain [21].

Psychological factors such as anxiety, depression, catastrophizing behaviours and pain-related fear have been demonstrated to be related to the experience of pain and physical function in people with a musculoskeletal injury [11, 22]. The association between resilience and musculoskeletal pain has been investigated, however, there are limitations in the published literature including inconsistency in the definition and classification of a musculoskeletal condition. For example, a recent systematic review used resilience theory to understand the potential outcomes after traumatic musculoskeletal injury, rather than explicitly assessing the resilience of participants. Whilst this paper is a systematic review, the included studies were of varied scientific rigour and the review itself examined a broad scope of participants with traumatic musculoskeletal injuries, including injuries as a result of non-mechanical injuries or events, such as burns and assaults [23]. Other studies have also included people who had sustained a traumatic spinal cord injury [24], which differs to pain exclusively experienced due to a musculoskeletal injury [25]. For example, there may be secondary musculoskeletal impairments such as contracture and spasticity with a brain or spinal cord injury, whereby the reason for pain and physical impairments are fundamentally linked to a disorder of the central nervous system. Others studies have included participants who had sustained injuries following a high energy trauma, or multiple injuries subsequent to a motor vehicle accident [23], with pain being multifactorial and not able to be directly or solely attributable to activity in the participants included in this systematic review. These inconsistencies in the literature limit the external validity of associations between resilience and MEP, which may also reflect differences in the mechanism, severity of injury and the types of activity that lead to pain.

Pain and its impact on activity are often assessed using self-reported questionnaires of pain interference which require a person’s ability to rate the severity of pain and degree of interference which pain has on their function [26, 27]. Commonly used scales such the Pain Interference Scale [26], assess general pain experience and combine the scores of activity interference and affective interference, making it difficult to ascertain the influence of pain associated with a pathology and its impact on physical function. Furthermore, it is difficult to discriminate between the person’s overall experience of pain and the relationship between pain and movement or functional activity. Establishing links between resilience and MEP can have significant clinical implications and may allow clinicians to determine treatment strategies used for recovery, which may include and combine physical rehabilitation or cognitive strategies. Exploring the relationship between MEP and resilience may therefore, help to streamline rehabilitation and allow clinicians to evaluate the importance of different treatments based on the relative weighting of barriers to rehabilitation such as a low level of resilience following a musculoskeletal injury. Rating pain severity during specific activities has not frequently been reported in the literature, with only 39% of published trials having included a measurement of MEP as a clinical outcome [28, 29]. Therefore, there is a need to directly examine MEP and its association with resilience with the aim of understanding how this relationship could be leveraged to improve functional outcomes and quality of life.

A preliminary search of MEDLINE, the Cochrane Database of Systematic Reviews, the Open Science Framework and JBI Evidence Synthesis was conducted and found no current or underway scoping reviews on the topic. Therefore, this scoping review aims to identify the volume of evidence, concepts and assessments that have investigated resilience in the experience of MEP and function after a musculoskeletal injury. Further, this review aims to identify and discuss gaps in the current knowledge base regarding resilience and MEP and indicate areas for future research to improve the assessment and outcome of people who have sustained a musculoskeletal injury.

## Methods

This scoping review was guided by the Joanna Briggs Institute (JBI) methodology for scoping reviews [30]. The JBI methodology has been influenced by the earlier work by Arksey and O’Malley and Levac and colleagues [31]. This study conforms to all Preferred Reporting Items for Systematic Reviews and Meta-Analyses (PRISMA) guidelines for Scoping Reviews (Additional File 1). The protocol for this scoping review (registration 10.17605/OSF.IO/J3XYH) can be found at the Open Science Framework Registries (https://osf.io/j3xyh).

### Search strategy

This scoping review utilised the Population, Concept and Context (PCC) format for inclusion [30] and operational definitions for each component of the PCC is provided in Table 1. To maximise the sensitivity of the search, a combination of keywords and index terms were used in literature searches to reflect the PCC. Literature searches were conducted from inception to 31st October 2021 on Cumulative Index to Nursing and Allied Health Literature (CINAHL) (Ebscohost), MEDLINE (OVID), APA Psychinfo (Ebscohost), Embase (OVID) and Web of Science (Clarivate). A full search strategy is provided in Additional File 2.

**Table 1:** Operational definitions for the population (musculoskeletal injury), concept (resilience) and context (movement-evoked pain or function) examined in the scoping review

PCC		Definition
Population	Adults with a musculoskeletal condition	A human adult, ≥ 18 years who has been diagnosed with or sustained a primary, mechanical or trauma-related injury/complaint of the musculoskeletal system. Injury of the musculoskeletal system is defined as any acute, persistent or chronic condition afflicting bones, muscles, ligaments, joints or connective tissue [4].
Concept	Resilience	A person’s ability to resist, withstand or recover, or adapt to stressors [12].
Context	Physical function OR Movement-Evoked Pain (MEP)	Physical function is defined as the ability of a person to perform tasks and activities of daily living that foster independence [32]. MEP refers to the experience of pain during activity, rather than pain experienced during rest [6, 7].

A Grey literature search was conducted on http://greylit.org using a basic keyword search of the term “resilience”. The first 100 records were eligible for screening and inclusion into the review.

Only journal articles available in full text and written in English were eligible for inclusion. Conference abstracts, editorials, poster presentations and books were not eligible for inclusion as these resources did not provide adequate detail to allow data extraction. Date ranges for publication were not imposed in order to capture a broad range of published literature on the topic.

### Study screening and selection

After searching the electronic databases, all search results were uploaded into EndNote (X9.3.3) and the duplicates removed. Before commencing screening, reviewers (ER and AG) completed training to ensure they had an in depth understanding of the research topic and the inclusion and exclusion criteria (Table 2) of the scoping review. A random selection of 100 search records was collated for pilot testing to ensure consistency in the screening and selection of records across reviewers. This process was consistent with the JBI protocol and identified any discrepancies between reviewers and ensured the inclusion and exclusion criteria was sufficient and clearly understood. Inter-rater agreement of 83% was achieved between the two independent reviewers within this pilot screening process.

**Table 2:** Inclusion ﻿and exclusion criteria used during screening for the population (musculoskeletal injury), concept (resilience) and context (movement-evoked pain or function) examined in the scoping review

	Inclusion	Exclusion
Population	• Adults (>18 years). • Examples for inclusion may include lower back pain, muscle strains, ligament tears, whiplash, knee osteoarthritis and fractures.	• Children or adolescents. • Caregivers of a person with an injury/disability. • Animals. • Conditions/disabilities characteristics of secondary musculoskeletal impairments that originated from systemic illness or inflammation (i.e.; post-polio syndrome, inflammatory arthritis). • Injuries to a body system other than the musculoskeletal system, such as the vascular system (i.e.; amputation due to diabetes) and central nervous system (i.e.; spinal cord injury, cerebrovascular accident, multiple sclerosis, complex regional pain syndrome). • • Cancer, pregnancy, polio.
Concept	• Validated assessment/outcome measure to examine of any type of resilience for human individuals (i.e.: physical resilience, psychological resilience or pain resilience).	• Resilience of an individual cell or system. • Resilience of a community. • Resilience of a caregiver. • Papers which examine single risk or protective factors which may be reflective of resilience (i.e.: self-efficacy).
Context	• Validated assessments/outcome measures which examine a movement-based task, dynamic movement or a functional task. • Validated assessments/outcomes which require a person to reflect on their function or disability.	• Surveys and questionnaires which do not examine function or movement (i.e.: satisfaction scale).
Other	• English language. • Full text.	• Non-English. • Full text unavailable.

Two independent reviewers screened the title and abstract of each article to determine their eligibility for inclusion. Articles were eligible for inclusion if they met each component of the inclusion criteria and did not have any criteria for exclusion (Table 2). Microsoft Excel (Version 2202 Build 16.0) was used to record the inclusion or exclusion of each title screened, using comments and coding. If the examination of resilience, a musculoskeletal diagnosis or physical test was unclear from the title or abstract, the record was included and progressed to a full-text review. A third reviewer (PC) was consulted if consensus was not able to be achieved between the two reviewers, with the third reviewer then deciding whether the article was to be included for analysis in the scoping review.

Hand searching of the reference lists of included studies was conducted to ensure additional studies eligible for inclusion were not missed during database searching.

### Data extraction and analysis

All records eligible for inclusion had their full text reviewed to extract relevant data to inform the scoping review’s question and objectives. Data was charted independently by one reviewer (ED), using a data extraction table (Additional File 3) adapted from the JBI Manual for Evidence Synthesis [30]. The following data was extracted from each record: publication details (author(s), year of publication); study location; study design; aims/purpose; study population (type of musculoskeletal injury, sample size, sex distribution and age); methodology; outcome measures (resilience scales and physical tests used); key findings and limitations of the study. Although previously described in the scoping review protocol, duration of symptoms was not recorded during data extraction as a number of studies examined surgical interventions and were interested in postoperative outcomes, rather than pre-operative symptoms.

Attempts were made to contact authors if any information required for data charting was not provided in the full text of the included article. Formulas in Microsoft Excel were used to calculate sums and averages for participant demographics and the musculoskeletal conditions examined. Microsoft Excel was also used to visually analyse this data, which is presented graphically (Fig. 2).

## Results

### Identification of studies

**Fig. 1:**
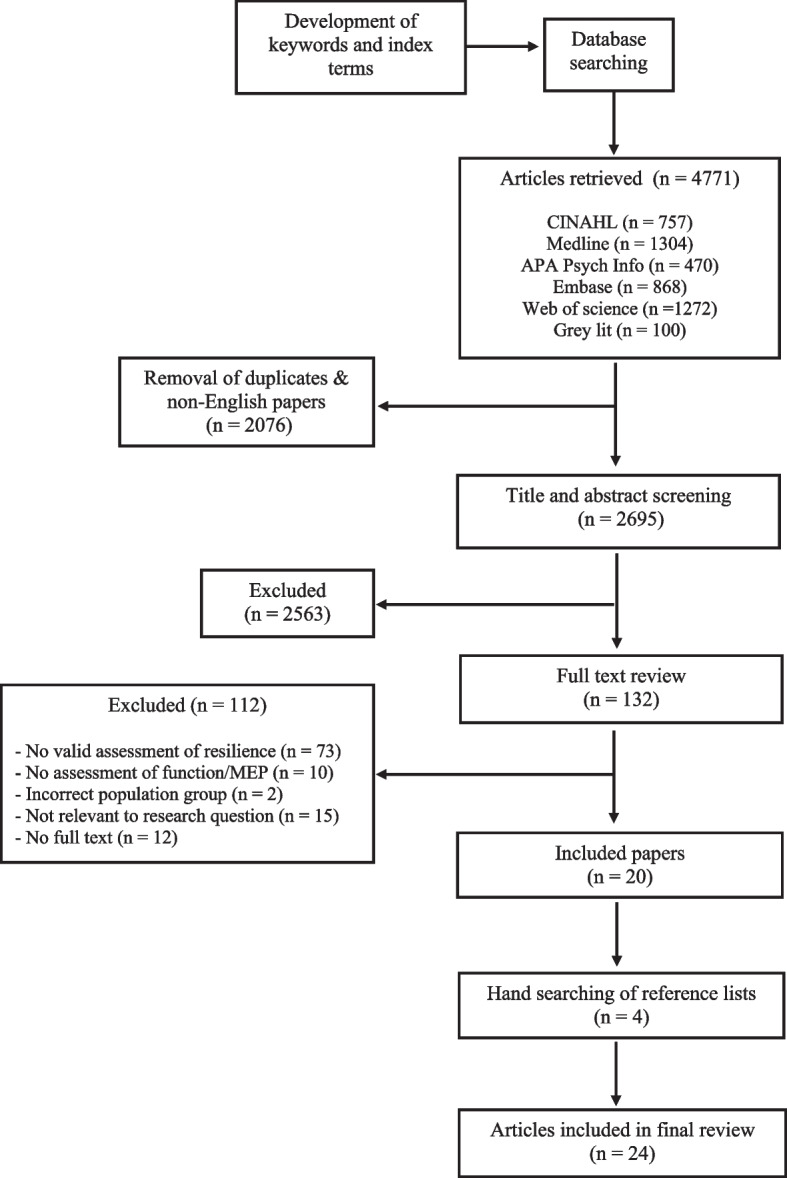
Preferred Reporting Items for Systematic Reviews and Meta-Analyses (PRISMA) flow chart

The PRISMA flowchart (Fig. 1) provides an overview of the study results. The initial search identified 4771 articles. After removing duplicates and non-English articles, 2695 papers underwent screening based on their title and abstract. After title and abstract screening 132 articles were eligible for full text review, from which 20 articles were included in the scoping review (Fig. 1). Hand searching the reference list of the included articles, identified 4 further papers for inclusion. A total of 24 articles were included in the final analysis. Of the 24 articles, 3 studies examined MEP and 21 studies examined function.

### Study characteristics

All of the 24 articles included were quantitative in design. Most studies (50%) were cross-sectional in design. There was a paucity of longitudinal research available which has examined the role of resilience in the experience of MEP and function after musculoskeletal injury.

### Data synthesis

Most studies included in the review (*n* = 15) reported that higher resilience was associated with lower MEP or greater physical function after a musculoskeletal injury. These findings were observed across a range of functional outcome measures and amongst a range of participant groups (Additional File 3), including those recovering from surgery [16, 33] or experiencing ongoing musculoskeletal pain [34–36]. Whilst inconclusive findings were often observed amongst common injury presentations, all articles examining participants after reverse total shoulder arthroplasty [37, 38] and hip fracture [39–41] reported consistent findings in support of resilience.

In contrast, 9 articles did not directly support a relationship between resilience and MEP or function. Several papers identified that resilience did not independently correlate with function, often with contextual or psychological variables moderating this relationship. For example, the relationship between psychological resilience and physical function was dependent on socioeconomic status and pain intensity [42], while pain acceptance was observed to moderate the relationship between resilience and functional outcomes [43].

### Study participants

The articles included in this review provided data on 5153 participants. The same pool of subjects were often used in studies with similar research teams [44–47], with these subject pools only included in the total population analysis once. Of these 5153 participants, 2059 were male, 3091 were female and 3 were unspecified. All articles included participants ≥18 years of age, with the average age of participants being 55.19 years. One study [48] did not collect data on the age of participants and acknowledged this to be a limitation of their work. Subsequently, the age of 108 participants included in this review is unknown.

Four papers [18, 42, 44, 45] provided socioeconomic demographics with the majority of participants reporting low socioeconomic status. Pooled data of 170 participants across three studies demonstrates that most participants (35.9%) had an annual income of <US $20,000. One paper [42] provided the monthly income for 143 participants, with approximately 80% of participants included in the study reporting an income less than or equal to 30,000 Nepalese rupees (approximately $225 USD).

**Fig. 2:**
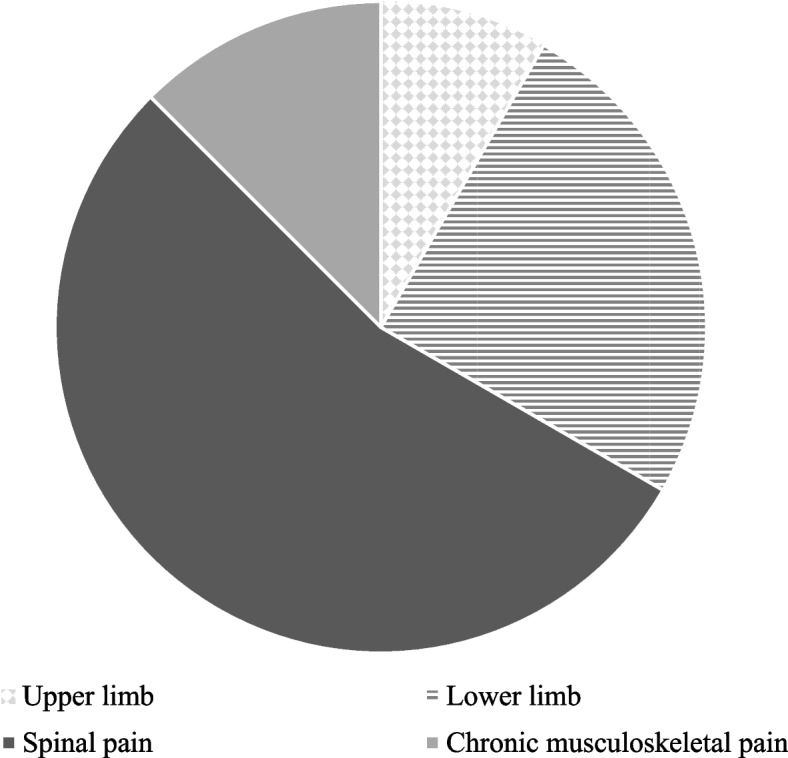
The distribution of musculoskeletal injury examined in all included papers (n = 24) which examined the role of resilience and movement-evoked pain or function

There were a range of musculoskeletal pathologies included in this review. MEP was examined in back pain (n = 2) and the lower extremity (knee pain) (n = 1), while function was examined in populations with upper extremity (shoulder arthroplasty) (n = 2), lower extremity (hip and knee surgery) (n = 6), back/spinal pain (acute and chronic spinal pain of various locations) (n = 10) and combined musculoskeletal pain sites (n = 3). Figure 2 depicts the pain sites of participants examined in articles of this review which examined the role of resilience in the experience of MEP or return to function. Eleven papers used a 0–10 scale to provided detail on single location pain intensity for participants. These studies assessed a total of 2426 participants and the pooled average pain intensity was 5.63.

Three articles examined ethnic minorities with a musculoskeletal injury including participants experiencing chronic musculoskeletal pain from rural Nepal [42], as well as knee osteoarthritis [18] and lower back pain amongst non-Hispanic individuals in the United States of America [44].

### Assessment tools

Four validated tools were identified and used to examine resilience. The most common resilience tools were the Brief Resilience Scale (BRS) (n = 10) and versions of the Resilience Scale (RS) (n = 10), followed by versions of the Connor Davidson Resilience Scale (CDRS) (n = 2) and the Pain Resilience Scale (PRS) (n = 2). Psychological resilience was the most common domain of resilience assessed, with only two articles [35, 45] assessing pain resilience in participants with back pain. Physical resilience, as defined in the current review, was not assessed in any of the included articles.

Physical function and MEP were assessed with a range of validated physical assessments, with the choice of assessment tool varying depending on the body area examined. The Roland-Morris Disability Questionnaire (RMDQ) (n = 5) and the Short Physical Performance Battery (SPPB) (n = 4) were the most common assessments tools used. The SPPB was used across a range of musculoskeletal conditions including back pain [44], knee osteoarthritis [18] and after hip fracture [41], whilst the RMDQ was used for participants with back pain [43, 46, 47, 49, 50].

Most studies used self-reported questionnaires to describe a participant’s functional ability, with only 7 studies utilising a physical assessment of functional performance on the day of data collection. Examples of physical assessments include the Short Physical Performance Battery (SPPB), the Lower Extremity Gains Scale (LEGS), Back Performance Scale (BPS) and step count measured with accelerometers (Additional File 3). Whilst many studies included a measure of pain severity, using the Numerical Pain Rating Scale (NPRS) or Visual Analogue Scale (VAS), pain assessment during movement was only examined in 3 studies (Additional File 3, Table 2).

### Concepts of resilience

Regardless of the assessment used to evaluate resilience, the conceptualisation of resilience also varied between papers, with six main themes being derived from analysis of the included papers. Sixteen papers referred to resilience as the ability to successfully adapt, recover, cope, manage or overcome stresses, adversity or challenging circumstances. Nine papers implied that resilience was timely, making reference to quick adaptation through phrases such as “quickly rebound”, “bounce back” and “spring back”. Six papers specifically commented that resilience was related to function, requiring someone to persist, manage or return to meaningful activities and function despite pain or repeated set-backs, such as pain flare ups. Two papers referred to the maintenance of equilibrium or avoidance of negative trajectories and three papers suggested resilience was associated with growth, flourishing and thriving. Three papers simply referred to resilience as a dispositional or constitutional variable and one paper did not describe their interpretation of resilience.

## Discussion

There is inconclusive evidence to confirm the relationship between resilience, MEP and function after a musculoskeletal injury. There are 15 studies which support a protective role of resilience when recovering from musculoskeletal injury, whilst 9 articles do not directly support this finding. While there was often diversity in the population groups and assessment tools used, which may impact findings, resilience appears to consistently have a protective role during recovery from hip fracture [39–41] and shoulder arthroplasty [37, 38].

The majority of studies in this review investigated resilience in older adults with age-related musculoskeletal conditions, such as osteoarthritis and chronic lower back pain. Although it was not an aim of this scoping review to include children or adolescents, there were no articles included in this review which had an average age of participants that was less than 40 years. This demonstrates a paucity of research examining resilience in younger people, limiting the ecological validity of the findings of this scoping review to young adults with musculoskeletal injury. Future longitudinal studies are required to investigate the role of resilience in the experience of musculoskeletal injury in younger adults to enhance the body of literature on the factors contributing to healthy aging and how resilience may change across the lifespan and in response to recovery from a musculoskeletal injury. Further studies are also required to investigate a range of musculoskeletal conditions, from relatively minor injuries that are expected to resolve quickly, to injuries that often lead to significant and long-term disability across an individual’s lifespan.

### Resilience tools

Currently, there is no available tool to explicitly assess the role of resilience when experiencing MEP due to a musculoskeletal injury. The available literature predominantly used scales that examine psychological resilience, to determine if the level of resilience could predict functional performance or movements which may evoke pain. The BRS and RS were the most utilised tools, as they were able to be administered quickly and were demonstrated to be reliable [51, 52]. Only two studies examined pain resilience and there was no published literature identified in this review which explored physical resilience in cohorts with a musculoskeletal injury. Despite the wide use of scales which examined psychological resilience, the items assessed may not be reflective of, or represent, MEP and function. For example, item three of the CDRS assesses spirituality. Although the importance of beliefs of fate and spirituality are believed to influence resilience [16], their relationship to physical performance is unclear. Similarly, the PRS requires a person to reflect on pain that may not be of a musculoskeletal origin or evoked by movement. Further, statements such as “when faced with intense or prolonged pain” does not reflect presentations which may be mild and quickly resolve. The Physical Resilience Scale is also phrased to allow a person to reflect on an injury related to age, and therefore excludes people who are not affected by age-related disease. The ambiguity of the available assessment tools highlights the need for tools that are more sensitive to being able to determine resilience to musculoskeletal pain which is movement based, varies in duration and is experienced by a broad range of ages.

This review confirmed a paucity of research which has examined MEP pain. Whilst many studies included a measure of pain to indicate disease severity, only three studies extended the measure of pain assessment during movement or activity. This finding is consistent with previous research on post-surgical clinical trials [29]. Further, the assessment tools used to examine MEP recorded outcomes from physical tests, however, the tools varied across the studies. Some assessment tools that objectively assessed functional movement were specific to a pathology such as the Back Performance Scale (BPS) [53], whilst others were general lower limb assessment tools, such as the SPPB, which assessed a range of populations including those with lower back pain, knee osteoarthritis and hip pathology. Studies which examined function used pathology specific questionnaires, such as the RMDQ, to determine how a patient’s own perception of their injury impacts their daily activity. Whilst pathology specific questionnaires may be more valid to assess function compared to the pain interference scale, which is not specific to a pathology, physical tests may be more appropriate to assess MEP as they assess an individual’s current level of physical performance rather than their anticipated performance. Further research is required to develop generic and condition-specific tools across a range of different musculoskeletal pathologies [54], to better differentiate MEP between acute and chronic presentations and enhance the identification of interventions with clinically relevant impacts on pain.

### Concepts of resilience

This review highlighted that resilience is a construct which can be conceptualised in many ways. Although various themes were identified in this review, the majority of studies agree that resilient individuals are able to cope and adapt to adversity. This is consistent with the definition of resilience presented in this review, which is an individual’s ability to maintain effective functioning by resisting, withstanding or recovering from stressors or adversity, including pain [12, 14]. Whilst some definitions consider a time or velocity component related to resilience, implied through phrases such as “spring back” and “bounce back”, the use of these phrases are largely open for interpretation and may be influenced by a range of contextual barriers such as injury severity, access to health care and socioeconomic status. For example, is someone considered more resilient if they bounce back from an ankle sprain and resume meaningful activity in two weeks compared to someone who has had a complex ankle surgery and returns to meaningful activity at 12 months? Some definitions of resilience propose an element of growth; however, this may introduce varied interpretations of whether growth is developed through an individual’s retrospective reflection on a previous adversity or whether it is a prospective observation made by others or the individual, in response to the current adversity. The concept of how resilience relates to MEP needs a consistent definition to allow a more accurate synthesis of information and to be able to guide future research.

Most studies considered the multidimensional nature of resilience and studied biopsychosocial factors, such as catastrophising [35, 45], depression [34, 55] or self-efficacy [33, 35, 56] that may impact a person’s resilience to the performance of physical tasks and the experience of pain. “Multisystem resilience” has been proposed to involve the assessment of a number of aspects which influence health including psychology, function and social history [57]. This review identified a study [58] that developed a resilience index, which included behavioural and psychosocial items to assess pain-related protective factors such as social support, active coping, positive and negative affect, waist-to-hip measures and tobacco use. The combined scores of these assessments gave a reflection of resilience and allowed stratification of participants into high and low resilience groups. Although this is not a validated measure of resilience and was therefore excluded from this review, it is reflective of the different components which combine to create multisystem resilience. Similarly, other studies [36, 57, 59] used clustering to group together risk factors and measures of resilience to demonstrate the combined effect of psychological variables on function. Whilst these studies were also excluded from the review as the independent effect of resilience on function was unable to be determined, these studies demonstrate the need for a formal and validated assessment tool that examines the multidimensional nature of resilience and explores its association with pain, which is influenced by biological, psychological and social factors.

The language used to describe and report on psychological traits in response to musculoskeletal injuries is inconsistent in the published literature screened during this review. “Psychological factors” and “psychological distress” were two commonly used phrases used in the reviewed literature, however, the combination of psychological factors assessed under these terms were numerous and varied between articles. For example “psychological factors” often included the combined assessment of catastrophising, anxiety, depression and fear avoidance beliefs, contrasting less frequently examined traits including personality types, anger and impulsivity. Similarly, “psychological distress” was often used to describe the combination of depression, pain disability and quality of life [60], while in other studies people with a combination of post-traumatic stress, depression, pain interference and activity restriction [61] were also described to experience “psychological distress”, therefore creating a heterogenous sample. A lack of standardised terminology and transparency in a number of abstracts resulted in these articles being included for full text review, however, a large majority of studies were subsequently excluded as it was apparent that resilience had not been assessed. There is a need for greater consistency in the use of language to describe psychological factors allowing synthesis of the published literature.

The need for greater language consistency is also depicted in the terms used to describe psychological status in response to musculoskeletal injuries. Resilience and grit and resilience and self-efficacy have frequently been used interchangeably; however appear to be different constructs [62]. Only a small number of studies identified in the current review explored the relationship between resilience and MEP, however, self-efficacy appears to have been more thoroughly investigated which was made apparent during title and abstract screening. Whilst resilience describes a person’s ability to resist, withstand or recover from stressors or adversity [12], it contrasts self-efficacy, which refers to a person’s self-belief or self-confidence that he/she can achieve a desirable outcome in response to a task that may be novel or difficult [63]. Similarly, grit refers to passion and perseverance for the attainment of long term goals [64, 65]. Theoretically, self-efficacy and grit can be present in the absence of a stressor and these traits may promote resilience and foster an individual’s ability to ‘bounce back’ or overcome stressful events [66, 67]. Whilst these traits do appear to be distinct from each other and is supported by literature which has included individual outcome measures to assess a combination of these traits [33, 35, 56, 59, 68], early research [69] describes five characteristics of resilience which include equanimity, perseverance, self-reliance, meaning, and existential aloneness. By definition, the characteristics of perseverance and self-reliance are also features of self-efficacy and grit, which may be a source of confusion and reflect the use of interchangeable terms in the literature. Further research is needed to clarify the definitions of each of these psychological constructs in relation to MEP, for which this scoping review has identified as an area of emerging research. Additionally, further research is required to disentangle the relationships between these psychological constructs and confirm if these traits are independent of one another or if they interact along a continuum to moderate or predict an individual’s performance and recovery trajectory [35, 36, 56, 70].

There were a selection of papers that were closely examined during full text review to determine if the population, concept and context used met the definitions of this scoping review. In one study grit was examined in a population of participants with Lower Back Pain (LBP) [64]. Whilst the examination of grit in a cohort of back pain appeared appropriate for this review, participants were actually instructed not to link their questionnaire responses to their experience of LBP. This therefore did not assess grit in relation to a musculoskeletal injury and excluded the paper from the review. Similarly, a virtual reality Sørensen test was assessed in participants with and without recurrent low back pain [71]. Whilst the Sørensen test is a well-known test of muscle endurance, it is used to predict first time episodes and recurrence of back pain [71], rather than predict functional performance. Five further studies outwardly fit the brief of the scoping review, however upon deeper examination were excluded due to inclusion of presentations not of a musculoskeletal origin [72], lack of detail to confirm pain of a musculoskeletal origin [73], use of an optimism outcome measure to assess resilience [74], absence of results reflecting the assessment of resilience [75] or use of assessments which were the combination of cognitive and functional domains [76]. These examples further demonstrate the need for transparency and consistent language in research, encouraging experts to establish clear definitions to guide research in musculoskeletal pain psychology which appears to be a developing topic area.

### Limitations

This scoping review aimed to explore the body of literature that examined the role of resilience in the experience of pain and function after a musculoskeletal disorder. Although systematic searching was performed across five key databases, there was a limit imposed on language and only one database was searched for grey literature. It is possible that the scoping review may have been more robust if the translation of non-English articles was feasible and if additional databases to search published and grey literature were included. A further limitation of this study is that the protocol for this scoping review did not undergo formal peer review. Whilst the protocol for this review was registered prior to commencement, it is possible that the peer review process may have provided feedback to enhance the methodological quality of the current review.

## Conclusion

This scoping review concludes that resilience in the context of MEP is an emerging construct. Greater knowledge of this relationship may ultimately influence the experience of those recovering from a musculoskeletal injury and allow clinicians to determine physical and cognitive treatment strategies to enhance recovery. While high quality research on pain resilience and physical resilience is still needed, there is inconclusive evidence to confirm if psychological resilience is associated with MEP and functional performance in older adults with a musculoskeletal injury. However, there is consistent evidence to support a protective role of resilience when recovering from shoulder and hip arthroplasty. It is acknowledged that factors such as pain intensity, financial income, race and ethnicity are variables that may moderate the relationship between resilience and MEP and should also be assessed in future studies. This scoping review also identified the need for future longitudinal research to examine younger adults to determine if multidimensional resilience may promote healthy musculoskeletal aging later in life.

## Supplementary Information

**Additional file 1.** The Preferred Reporting Items for Systematic reviews and Meta-Analyses extension for Scoping Reviews (PRISMA-ScR) Checklist completed for the conducted scoping review.

**Additional file 2.** Search strategy used for database searching. Example of a full search strategy (Medline) used for data collection in the scoping review. The search strategy includes a combination of key words and index terms.

**Additional file 3.** Data charting table and extracted data. The extracted data obtained for each included paper of the scoping review. Data has been extracted according to the data charting table adapted from the JBI Manual for Evidence Synthesis.

## Data Availability

The dataset supporting the conclusions of this article is included within the article (and its additional files).

## Abbreviations

ADL: Activities of Daily Living
BPS: Back Performance Scale
BRS: Brief Resilience Scale
CRDS: Connor-Davidson Resilience Scale
CINAHL: Cumulative Index to Nursing and Allied Health Literature
DALYs: Disability-Adjusted Life Years
JBI: Joanna Briggs Institute
LBP: Lower Back Pain
LEGS: Lower Extremity Gains Scale
MEP: Movement-evoked pain
NPRS: Numerical Pain Rating Scale
PRISMA: Preferred Reporting Items for Systematic Reviews and Meta-Analyses
PCC: Population, Concept and Context
PRS: Pain Resilience Scale
RMDQ: Roland-Morris Disability Questionnaire
RS: The Resilience Scale
SPPB: The Short Physical Performance Battery (SPPB)
VAS: Visual Analogue Scale
