# Supplementary material for: A scoping review on the methods of assessment and role of resilience on function and movement-evoked pain when experiencing a musculoskeletal injury
Source: BMC Musculoskelet Disord. 2022 Dec 15;23:1097. doi: 10.1186/s12891-022-06058-2 (PMC9753293; doi:10.1186/s12891-022-06058-2)
Supplement: Supplementary file 2 — Additional file 2. Search strategy used for database searching. Example of a full search strategy (Medline) used for data collection in the scoping review. The search strategy includes a combination of key words and index terms. [file 12891_2022_6058_MOESM2_ESM.docx]

**Additional File 2:** Example of a full search strategy (Medline) used for data collection in the scoping review.

Adaptation, Psychological/ OR Resilience, Psychological/ OR (reslien* OR hardiness OR grit OR mental toughness OR psychological factor*).ti,ab. AND Functional status/ OR Motor Activity/ OR (physical performance OR function* OR motor activit* OR movement OR activit* OR mobility OR ability OR disability OR movement?evoked pain OR activity?related pain OR recover* OR outcome*).ti,ab. AND Musculoskeletal System/ OR (musculoskeletal OR musculoskeletal diagnosis OR athletic injur* OR upper extremit* OR lower extremit* OR neck OR back) .ti,ab. AND Musculoskeletal Pain/ OR Pain, Postoperative/ OR Pain/ OR (pain*).ti,ab.
