# Supplementary material for: A scoping review on the methods of assessment and role of resilience on function and movement-evoked pain when experiencing a musculoskeletal injury
Source: BMC Musculoskelet Disord. 2022 Dec 15;23:1097. doi: 10.1186/s12891-022-06058-2 (PMC9753293; doi:10.1186/s12891-022-06058-2)
Supplement: Supplementary file 3 — Additional file 3. Data charting table and extracted data. The extracted data obtained for each included paper of the scoping review. Data has been extracted according to the data charting table adapted from the JBI Manual for Evidence Synthesis. [file 12891_2022_6058_MOESM3_ESM.docx]

**Table 1:** Extracted data for papers assessing resilience and function in musculoskeletal populations.

| **Authors** | **Location** | **Study design*** | **Study population (Injury diagnosis, sample size, gender, age**)** | **Aims of study** | **Methodology** | **Outcome measures***** | **Important results** | **Limitations of study** |
| --- | --- | --- | --- | --- | --- | --- | --- | --- |
| Dombrowsky et al. (2021). | USA | RR | Primary reverse total shoulder arthroplasty (r TSA) indicated for cuff tears or arthropathy and osteoarthritis with glenoid bone loss.  N = 73.  Females = 43  Males = 30  Age = 70 | To explore the relationship between resilience and patient reported and functional outcomes after rTSA. | rTSA surgery was performed by a single surgeon. Participants completed phone surveys for all outcomes and were stratified based on resilience level. | - BRS - The American Shoulder and Elbow Surgeon score (ASES) - The Penn shoulder score (Penn) - The Single Assessment Numerical Evaluation (SANE) scores | - Non-significant differences (p = 0.12) for the Penn between the high and low resilience groups. - Significant results were observed for the ASES (activities of daily living) (p = 0.04) and SANE scores (self-report of normal function) (p = 0.02) between the high and low resilience groups. - Patients classified with higher resilience were more likely to report higher outcome scores. | - Generalisability impacted by inclusion of a single surgeon and single institution. |
| Tokish et al. (2017). | USA | PS | Total Shoulder Arthroplasty (TSA).  N= 70  Females = 51  Males = 19  Age= 65 | To study correlations between the Brief Resilience Scale and self-reported shoulder function after TSA. | TSA surgery was performed. Participants completed standardized rehabilitation protocols implemented by a physiotherapist. Participants were followed up for a minimum of 2 years and completed outcome measures at their final review. Participants were stratified into groups based on their resilience level. | - BRS - ASES - SANE - Penn | - Post operative BRS scores significantly correlated with ASES, Penn and SANE scores (< 0.004). - The low resilience group had significantly lower scores on the Penn (34 points lower) and SANE (40 points lower) compared to the high resilience group. | - Psychological variables were not assessed as potential confounders. - Results may have lower generalisability to male participants. |
| Silverman et al. (2020). | USA | PCS | Primary arthroscopic management for intra-articular hip pathology (including FAI, labral tear, internal snapping hip both in native and artificial joints, and femoral dysplasia).  N = 40  Females = 17  Males = 23  Age = 40 | To explore the relationships between resilience and patient reported outcome measures (PROMs). | Patients were recruited at surgical appointment and completed survey questionnaires before surgery and at post-op appointments. | - BRS - Modified Harris Hip Score (mHSS) - Visual Analogue Scale (VAS) - Hip Outcome Score Activity of Daily Living Scale (HOS-Daily) - Hip Outcome Scale-Sports (HOS-Sports). | - Significant relationships between resilience and all the PROMs (except the Hip Outcome Scale- Sports) both preoperatively and at 6 months postop (p < 0.05) between the low resilience and high resilience groups. - BRS scores showed a significant positive correlation with the mHHS and HOS-Daily - Trend for those with normal and high resilience to recovery early. | - Small sample size (was originally a pilot study). - Surgery was performed by a single surgeon. - Binary measures were used to assess anxiety and depression. |
| Resnick et al. (2019). | USA | PCS | Surgical repair of a hip fracture.  N = 172  Female = 86  Males = 86  Age = 81 | To examine associations between demographics and psychological variables on resilience, function and exercise post hip fracture. | A face-to-face structured interview obtained demographic and health information and blood samples. At two, six and twelve months post hip fracture, participants were interviewed again repeat data collection. | - Modified version of the 25-Item Resilience Scale (RS) - Lower Extremity Gain Scale (LEGS) - Short Physical Performance Battery (SPPB) - Grip strength (dynamometer) - Modified form of the Functional Status Index - Modified version of the Older Americans Resources and Services Instrument (OARS) - Exercise subscale of the Yale Physical Activity Survey (YPAS) - Total areas of pain and a sum of pain intensity (0-10). - Blood test collections. | - Resilience, age, cognition, social activity, and pain intensity were directly associated with function. - Genetic variability was indirectly associated with function through resilience. - Resilience, age and cognition were directly associated with exercise. - Genetic variability was indirectly associated with exercise through resilience. | - Study participants did not regularly participate in exercise. Results reflective of exercise may be associated with ongoing physiotherapy after surgery. |
| Resnick et al. (2018). | USA | PCS | Surgical repair of a hip fracture.  N = 258  Female = 133  Males = 125  Age = 81 | To test factors influencing physical function and performance at 2 months post hip fracture, between men and women. | Participants admitted to hospital with hip fracture were screened and enrolled. Female enrolment was matched to males. Demographic data was obtained at baseline and all other measures were obtained at 2 months post hip fracture. | - Modified version of the 25-Item RS. - Number of pain sites. - Measures of body composition. - YPAS - LEGS - SPPB - Grip strength (hand dynamometer) - Modified Charlson scale - Modified Mini-Mental State. | - Resilience was not associated with pain. - Resilience was associated with physical activity, performance and function among women only. - Resilience was only associated with physical activity in men. - Those that were more resilient spent more time in physical activity. | - Did look at lean body mass, however did not examine strength or muscle quality which could impact physical function and performance. |
| Crijns et al. (2019). | USA | CSS | Hip or knee osteoarthritis +/- arthroplasty (30% hip OA, 60% knee OA, 10% hip and knee OA. 45% post arthroplasty)  N = 108  Female = 74  Male = 34  Age = not recorded | To determine correlations between psychological traits on pain intensity and functional limitations amongst participants with symptomatic hip or knee OA. | Patients visiting an orthopaedic surgeon were enrolled and completed anonymous questionnaires. | - BRS - Patient Activation Measure (PAM) - PSEQ-2 - Hip Disability and Osteoarthritis Outcome Score, Junior (HOOS, JR) - Knee Injury and Osteoarthritis Outcome Score, Junior (KOOS, JR) - NPRS | - The BRS was not independently associated with the KOOS, HOOS or NPRS. - The BRS, PSEQ-2 and PAM correlated with one another. | - Age was not recorded during data collection. |
| Magaldi et al. (2019). | USA | PCS | Primary elective total knee arthroplasty (TKA).  N = 153  Female = 79  Male = 74  Age = 68 | To examine the relationship between resilience and patient-reported outcomes following TKA. | Patients were recruited at preoperative education sessions and provided demographics and completed questionnaires. Participants were then contacted to complete follow up questionnaires at 3 months and 12 months post-surgery. | - BRS - The Knee Injury and Osteoarthritis Outcome Score JR (KOOS-JR) - The PROMIS-10 Global Health Assessment. | - Resilience measured prior to TKA correlated significantly with outcomes of quality and life (mobility, self-care, usual activities, pain) and mental health quality of life (anxiety/depression), at 3 and 12 months, but not outcomes relating to knee-specific function. - A significant decrease in resilience was observed amongst participants with poor KOOS-JR outcomes at 12 months, suggesting that stressful events can cause transient changes in resilience. | - Over-reliance on self-reported measures. |
| Slepian et al. (2020). | USA` | LCS | Self- reported acute lower back pain.  N = 343  Female = 211  Male = 129 Unspecified gender = 3  Age = 40 | To determine if catastrophising and pain resilience will enhance the predictive utility of the fear avoidance model. | Participants were recruited from an online marketplace and received low monetary compensation at the end of the study. Patients completed self- reported pain, psychological and functional questionnaires at baseline, 1 month and 3 months, using Qualtrics. | - Pain Resilience Scale (PRS) - Demographics (race, gender, age, employment) - Pain Catastrophizing Scale (PCS). - Tampa Scale of Kinesiophobia (TSK). - Pain Self-Efficacy Questionnaire (PSEQ) - The McGill Pain Questionnaire (MPQ) Short-Form-II. - VAS - PROMIS Depression Scale - PROMIS Physical Dysfunction Scale | - The effect of resilience on long term function was moderated by self-efficacy and Kinesiophobia. - Resilience did not have a direct effect on pain intensity or physical dysfunction. - Pain resilience was indirectly and negatively associated with pain intensity, physical dysfunction and depressive symptoms. | - Potential for reporting inconsistencies as back pain was self-reported. Diagnosis was not based on imaging or objective measures. |
| Jegan et al. (2017). | Germany | PCS | Chronic lower back pain (CLBP) or Chronic Widespread Pain (CWP).  N = 423  Female = 244  Male = 179  Age = 57 | To examine the longitudinal impact of resilience and coping resources on disability. | Patients were recruited and had demographic data, pain characteristics and potential risk and protective factors measured at baseline. Patients had disability remeasured again at 12 months. | - RS - The Pain Characteristics subscale of the German Pain Questionnaire. - Pain drawings. - Graded Chronic Pain Scale (GCPS) (pain intensity and pain-related disability). - Hospital Anxiety and Depression Scale (HADS) - German version of the somatization subscale of the Symptom Checklist-90-R (SCL-90-R). - The Coping Resources for Back Pain Questionnaire (CRBPQ). - West Haven-Yale Multidimensional Pain Inventory (MPI-D) | - Resilience did not predict follow-up disability. Small cross sectional associations were identified between resilience and disability (Cohens 0.003); however this correlation did not carry over to longitudinal multivariate analysis. | - Examined participants with long term pain – 60% of participants had pain for >5 years. Limits external validity of findings. |
| Wilks et al. (2009). | USA | CSS | Lower back pain.  N= 164  Females = 87  Male = 77  Age = 45 | To determine the mediating effects on the relationship between age and lower back pain. | Participants were recruited from fitness facilities. Surveys were completed by participants and collected by staff at the participating facilities. | - RS - 6-item Psychological Vulnerability Scale - 3-item Avoidance Coping Scale - 4-item Brief Resilient Coping Scale - Owestry Disability Index (ODI) | - Resilience was not associated with ODI. - The only factor that was significantly inversely correlated with ODI was avoidance coping. | - Had to consult primary data analysis (Guillory et al., 2008) to determine methodology and patient demographics. |
| Ruiz-Párraga et al. (2015). | Spain | CSS | Chronic back pain (Cervical 52%, Thoracic 32%, Lumbar 68 % and Sacral 52 %)  N = 592  Females = 421  Males = 171  Age = 46 | To explore the structure of the Resilience Scale and investigate its validity in chronic pain adjustment. | Participants were referred to the study by physicians and physiotherapists. After screening, participants attended an appointment to complete a battery of questionnaires during a semi-structured interview with a psychologist (approximately 1.5 hours). | - RS adapted to Chronic Pain Patients (RS-18) - Anxiety Sensitivity Index (ASI) - PCS - Fear-Avoidance Beliefs Questionnaire (FABQ). - Pain Vigilance and Acceptance Questionnaire (PVAQ) - Chronic Pain Acceptance Questionnaire (CPAQ) - Composite Pain Intensity Index - HADS - The Impairment and Functioning Inventory (IFI) | - Medium effect sizes were found between the RS-18 and functional impairment. - Resilience was independently associated with functional impairment and daily functioning. - RS-18 was positively associated with pain acceptance and higher levels of daily function and negatively related to functional impairment. | - Attempts were not made to control for use of pain interventions (e.g. medication, physiotherapy). |
| Ramírez-Maestre et al. (2017). | Spain | CSS | Acute back pain (Cervical 44.8%, Thoracic 26.1%, Lumbar 21.1%, Sacral 28.9% and leg below knee 3%).  N = 232  Females = 157  Males = 75  Age = 45 | To investigate the role of psychological variables in acute pain disability. | Each participant participated in a semi-structured interview with a psychologist to obtain demographic, social, or medical history data. A battery of questionnaires was also completed by each participant. | - RS - ASI - PCS - FABQ - HADS - NPRS - Roland-Morris Disability Questionnaire (RMDQ). | - No correlations were found between resilience and disability. - Both fear- avoidance and pain intensity were associated with disability. | - Reliance on self-reported outcome measures. |
| Ramírez-Maestre et al. (2014). | Spain | CSS | Chronic spinal pain.  (Cervical 53.4%, Thoracic 34.1%, Lumbar 36.9%, Sacral 63.7% and leg below knee 35.4%).  N = 686  Female = 405  Male = 281  Age = 45 | To investigate the role of resilience and risk factors in the experience of chronic pain. | Participants each had a semi-structured interview with a psychologist to obtain demographic, social, or medical history data. A battery of questionnaires was also completed by each participant. | - RS - Acceptance & Action Questionnaire (AAQ) - PCS - Pain Vigilance and Awareness Questionnaire (PVAQ) - FABQ - Chronic Pain Acceptance Questionnaire (CPAQ) - HADS - RMDQ - IFI - NPRS | - Resilience did not have a direct relationship with functional impairment. - Resilience had a high correlation with pain acceptance. - Pain acceptance had significant, negative and moderate correlation with impairment. The higher the pain acceptance, the lower the functional impairment and pain intensity. | - Reliance on self-reported outcomes. |
| Ramírez-Maestre et al. (2012). | Spain | CSS | Chronic spinal pain.  (Cervical 52.5%, Thoracic 32.1%, Lumbar 88.6%, Sacral 57.5% and leg below knee 28.4%).  N = 299  Female = 161  Male = 138  Age = 44 | To analyse relationships between resilience, acceptance, coping and adjustment in a cohort of chronic spinal pain. | Participants completed a semi-structured interview with a psychologist to collect socioeconomic, demographic, pain and medical data. Participants also completed a battery of questionnaires. | - RS - CPAQ - The Vanderbilt Pain Management Inventory - Passive and active dimensions of coping - HADS - The Impairment and Functioning Inventory. - NPRS | - Bivariate analysis identified resilience was significantly negatively associated with acceptance, active coping, functional impairment, anxiety and depression. - Acceptance of pain influenced functional impairment and functional status. - Resilience had a direct and positive effect on pin acceptance and coping. |  |
| Esteve et al. (2017). | Spain | PCS | Acute back pain (Cervical 45%, Thoracic 22%, Vertebral-lumbar 37% and Lumbar-renal 21%, Sacral 29%).  N = 232  Females = 157  Male = 75  Age = 45 | To investigate the predictive power of resilience and vulnerability factors in acute back pain related disability. | Participants were recruited and assessed on five occasions during semi-structured interviews (45 mins). The first assessment was conducted when pain duration was less than three months, and subsequently at six month intervals. Each interview collected demographic, social, medical history data and battery of questionnaires. | - RS - ASI - FABQ - NPRS - HADS - RMDQ - Demographic and pain variables (age, sex, marital status, education, work status, pain duration and location, medications and other medical treatments). | - Resilience was not associated with pain-related disability over time. - Only pain intensity and pain-related disability were predictors of greater disability during follow up. - Resilience showed a small negative correlation with depression. | - High attrition rate - Reliance on self-reported measures |
| Coronado et al. (2021). | USA | RCT | Patients post laminectomy (with or without fusion) for a degenerative lumbar condition (spinal stenosis, spondylosis with or without myelopathy, and degenerative spondylolisthesis).  N = 248  Females = 126  Males = 122  Age = 62 | To examine the association of early postoperative resilience and self-efficacy on 12 month outcomes after lumbar spine surgery. | Participants completed a preoperative assessment and medical records were reviewed to collect demographics, clinical and medical information. A baseline assessment was completed 6 weeks after surgery, and participants were randomly assigned to an intervention arm (cognitive-behavioural-based physical therapy or education). Reassessment of baseline measures was conducted 12 months after surgery. | - BRS - PSEQ - PROMIS-PF - ODI - NPRS - Accelerometer activity counts for physical activity. | - Resilience was moderately correlated with all patient reported outcomes (range of r=49 to 0.34 and 0.34 to 0.37, p = <0.001) - Higher resilience and pain-self-efficacy were associated with higher physical function and social participation and lower pain interference, disability and back pain. - Baseline resilience was an important determinant of physical function at 12 months post op. | - Did not consider other psychological traits as confounding variables in analysis. |
| Ahmed et al. (2019). | USA | PCS | Neck (23%) and back pain (77%) with or without radiculopathy.  N = 180  Females = 97  Males = 83  Age = 53 | To examine relationships between resilience, pain self-efficacy and disability in patients with neck or back pain. | Patient were recruited at an orthopaedic appointment. Patients completed a survey of demographic information and questionnaires for required outcome measures. | - BRS - PSEQ-2 Short Form - Neck Disability Index (NDI) - ODI | - BRS was strongly negatively correlated with NDI (r = -0.61, p <0.0001) and ODI (r= -0.34, p <0.0001). - Low resilience and low pain self-efficacy were both independently associated with greater functional disability in neck and low back pain patients. | - 15 participants were excluded due to missing data. - Short form of PSEQ used. Other versions exist. |
| Chen & Jackson (2018). | China | CSS | Chronic back pain (cervical 52%, lumbar, 42%, less localized back pain 6%).  N = 307  Females = 189  Males= 118  Age = 53 | To determine if resilience and the appraisal of pain is associated with coping and pain-related dysfunction. | Participants were recruited via print advertisements and referrals. Participants were given information on the study, completed the test battery and received monetary compensation for participation. | - 10-item Connor-Davidson Resilience Scale–Chinese (CDRS-C) - Pain Appraisal Inventory–Short-Form–Challenge (PAI-SF-C) - PSEQ - Coping Strategies Questionnaire–Catastrophizing subscale (CSQ-C) - Chronic Pain Grade (CPG) - Multidimensional Pain Inventory Screening Chinese– Affective Distress subscale (MPI-sC) - Centre for Epidemiologic Studies Depression Scale (CES-D). | - Resilience was associated with lower pain-related dysfunction (p <0.05). - The path from resilience to pain-related dysfunction was also influenced by catastrophising and self-efficacy. | - Measure of dysfunction used is a combined score of pain intensity and interference. |
| Newton-John et al. (2014). | Australia | CSS | Chronic pain (Primary pain site Back 69.3%, upper limb 17.8%, lower limb 7.9%, Ankle/foot 2% or other 2%).  N = 101  Female = 57  Male = 44  Age = 43 | To enhance understanding of how resilience is involved in coping and adjustment to chronic pain. | Participants were screened and enrolled at a pain clinic appointment. Participants completed a series of questionnaires in the waiting room (approximately 30 mins). | - BRS - NPRS - PSEQ - TSK - PCS - RMDQ - Depression, Anxiety and Stress Scale (DASS) - OSLO-3 Social Support Scale. | - BRS showed significant negative relationships with pain-related disability, pain intensity and fear of movement. - Resilience was associated with a greater likelihood of attending work, with more resilient individuals more likely to continue working despite pain. | - 81% of participants in this study were being managed under the workers-compensation scheme which limits the external validity of findings to non-compensable patients. |
| Sharma et al. (2018). | Nepal | CSS | Chronic musculoskeletal pain (multiple sites 60%, low back and pelvis 32%, knee 30%, shoulder 8%, neck and upper extremity 8%, other 5%).  N = 143  Female = 93  Males = 50  Age = 47 | To examine the associations between socioeconomic and psychological factors and measures of function in individuals with chronic pain from rural Nepal. | Patients seeking care at a tertiary care hospital or those in a rural community of Nepal were recruited. After screening, participants completed a face-to-face interview to provide demographic information and complete questionnaires. | - 2-item CDRS - Pain drawing - NPRS - PCS - Beck Depression Inventory (BDI) - Nepali version of the Patient-Specific Functional Scale (PSFS). | - Resilience and catastrophising did not make statistically significant independent contributions to physical function. - Pain intensity and income moderated the association between resilience and physical function. - Resilience was negatively associated with depression in people with lower income. | - Limited external validity. Results may only be reflective of the ethnic group examined. - Etiology of pain was not considered which may influence coping and pain adjustment. |
| Ruiz-Párraga et al. (2012). | Spain | PCS | Chronic musculoskeletal pain (38% low back pain, 32% spinal disc disease or cord clamping, 30% cervical pain).  N = 300  Female = 203  Male = 97  Age = 47 | To evaluate the validity and reliability of the resilience scale in patients with chronic musculoskeletal pain. | Participants completed a series of questionnaires whilst waiting to be seen by a physician. Participants were contacted by phone six months later to collect further data. | - RS - Questionnaire on demographics and medical history. - PCS - CPAQ - Vanderbilt Pain Management Inventory (VPMI) - HADS - Pain Anxiety Symptoms Scale (PASS) - RMDQ - The Impairment and Functioning Inventory (IFI) - NPRS | - Findings support a protective role of resilience. - Medium effect sizes were found between RS and disability (RMDQ -0.39) and functional impairment (IFI impairment subscale -0.46). - Large effect size was found between RS and daily functioning (IFI functioning subscale 0.54). | - Pain interventions were not controlled. |

*Abbreviations for common study designs: RR: Retrospective Review, PS: Prognosis study, PCS: Prospective cohort study, CSS: Cross sectional study, RCT: Randomised control trial, LCS: Longitudinal cohort study; **Age reported as mean of sample, in years. *** Abbreviations for commonly used outcome measures in studies: BRS: Brief Resilience Scale, PRS: Pain Resilience Scale; RS: Resilience Scale, CDRS: Connor-Davidson Resilience Scale; FABQ: Fear Avoidance Beliefs Questionnaire; NPRS: Numerical Pain Rating Scale; HADS: Hospital Anxiety and Depression Scale; RMDQ: Roland-Morris Disability Questionnaire; ODI; Owestry Disability Index; NDI: Neck Disability Index; SPPB: Short Physical Performance Battery, BPS: Back Performance Scale; CPG: Chronic Pain Grade, PCS: Pain Catastrophizing Scale; PSEQ: Pain Self-Efficacy Questionnaire.

**Table 2:** Extracted data for papers assessing resilience and movement-evoked pain (MEP) in musculoskeletal populations.

| **Measures of Movement-Evoked Pain (MEP)** | | | | | | | | |
| --- | --- | --- | --- | --- | --- | --- | --- | --- |
| **Authors** | **Study location** | **Study design*** | **Study population (Injury diagnosis, sample size, gender, age**)** | **Aims of study** | **Methodology** | **Outcome measures***** | **Important results** | **Limitations of study** |
| Palit et al. (2020). | USA | CSS | Chronic lower back pain (CLBP).  N = 60  Female = 34  Male = 26  Age = 68 | To determine if pain resilience moderates the relationship between negative pain beliefs and functional performance for older adults with cLBP. | Participants attended two laboratory sessions (2.5-3hrs), 1 week apart. During session 1, participants completed demographic and medical assessment, anthropometric tests, psychological questionnaires and functional performance measures. Additional psychological questionnaires completed at home between each formal session and during the second laboratory session. Participants received monetary compensation for their involvement. | - PRS - BMI - PCS - FABQ - Back Performance Scale (BPS) - VAS after completion of each of tasks on the BPS. | - Resilience did not moderate functional performance. - Higher fear avoidance and catastrophising was associated with more MEP in participants with low pain resilience. - Pain resilience moderated the relationships between fear-avoidance beliefs, catastrophic thoughts about pain and MEP. - Individuals with higher fear-avoidance and pain catastrophizing reported greater MEP, however, this was attenuated in individuals with higher levels of pain resilience. | - Small sample size - Assessment of older adults (>60 years). |
| Morais et al. (2021). | USA | CSS | CLBP.  N = 60  Females = 34  Males = 26  Age = 68 | To determine the effect of race differences on resilience factors and pain-related outcomes for older adults with CLBP. | Participants attended an appointment to collect demographic, health, anthropometric psychological and functional data.  Participants also completed questionnaires from home. Participants received monetary compensation. | - BRS - Gratitude Questionnaire (GQ-6) - PROMIS Emotional Support Scale - SPPB - VAS after completion of each item of the SPPB. | - Resilience accounted for 31% of the variance in MEP (p = 0.02). - There was significant interaction between race and resilience. - Higher resilience was associated with lower MEP among Non-Hispanic whites (NHWs); however, this was non-significant and in the opposite direction for NHBs. - Significant interaction between gratitude and SPPB function. | - Unequal distribution of participants between groups ( NHB n = 15 and NHW n = 45). - Assessment of older adults (>60 years). |
| Bartley et al. (2019). | USA | CSS | Participants with or who are at risk of KOA.  N = 201  Female = 123  Male = 78  Age= 58 | To investigate associations between resilience, clinical pain, and functional performance in people with KOA and determine if race/ethnicity moderate these relationships. | Telephone screening obtained sociodemographic and health data. One week later, participants attended a health assessment to collect health data, complete functional measures and be assessed by a rheumatologist. After the health assessment, participants completed a series of questionnaires. | - BRS - LOT-R - Positive Affect and Well-Being (PAW)–Short Form - Multidimensional Scale of Perceived Social Support - PANAS - GCPS - SPPB - VAS after completion of each item of the SPPB. | - Higher resilience was associated with lower pain and disability and higher physical functioning. - Pattern for those with higher resilience to have lower pain intensity, disability, MEP and higher physical functioning (p > 0.08). - No differences across race/ethnicity in resilience, but significant differences for pain and function. - NHBs reported greater pain, disability, physical function and MEP compared with NHWs. | - Subgroup analysis was not performed for those with KOA and those at risk of KOA. Majority of participants had pain >5 years. - Assessment of older adults (>45 years). |

*Abbreviations for common study designs: RR: Retrospective Review, PS: Prognosis study, PCS: Prospective cohort study, CSS: Cross sectional study, RCT: Randomised control trial, LCS: Longitudinal cohort study; **Age reported as mean of sample, in years. *** Abbreviations for commonly used outcome measures in studies: BRS: Brief Resilience Scale, PRS: Pain Resilience Scale; RS: Resilience Scale, CDRS: Connor-Davidson Resilience Scale; FABQ: Fear Avoidance Beliefs Questionnaire; NPRS: Numerical Pain Rating Scale; HADS: Hospital Anxiety and Depression Scale; RMDQ: Roland-Morris Disability Questionnaire; ODI; Owestry Disability Index; NDI: Neck Disability Index; SPPB: Short Physical Performance Battery, BPS: Back Performance Scale; CPG: Chronic Pain Grade, PCS: Pain Catastrophizing Scale; PSEQ: Pain Self-Efficacy Questionnaire.
